# New insight into the role of the ADAM protease family in breast carcinoma progression

**DOI:** 10.1016/j.heliyon.2024.e24805

**Published:** 2024-01-19

**Authors:** Sepideh Aliniaye Navasatli, Saeed Niazi Vahdati, Tahura Fayeghi Arjmand, Marjan Mohammadi far, Hossein Behboudi

**Affiliations:** a Institute of Biochemistry and Biophysics, Department of Biochemistry, University of Tehran, Tehran, Iran; b Department of Medical Biotechnology, Faculty of Advanced Medical Sciences, Tabriz University of Medical Sciences, Tabriz, Iran; c Department of Biology, Medicinal Plants and Drugs Research Institute, Shahid Beheshti University, Tehran, Iran

**Keywords:** A disintegrin and metalloprotease, Breast cancer, Metastasis, Treatment

## Abstract

Protease and adhesion molecules play a very emphasized role in the occurrence or progression of metastasis in many types of cancers. In this context, a molecule that contains both protease and adhesion functions play a crucial role in metastasis. ADAMs (a disintegrin and metalloprotease) are molecules with this special characteristic. Recently, a lot of attention has been attracted to various ADAM molecules and researchers have tried to elucidate the role of ADAMs in breast cancer occurrence and progression. Disrupting ADAMs protease and adhesion capabilities can lead to the discovery of worthy therapeutic targets in breast cancer treatment. In this review, we intend to discuss the mechanism of action of various ADAM molecules, their relation to pathogenic processes of breast cancer, and their potential as possible targets for breast cancer treatment.

## 1 Introduction

Breast cancer is the most common malignancy and the second leading cause of cancer-related death in women worldwide [1]. Hormone blockers, chemotherapy, and targeted EGF receptor 2 antibodies altogether have moved forward the post-surgical survival of breast cancer (BC) patients. However, patients long-term survival has remained poor due to the high chance of distant metastasis formation [2]. One hallmark of the progressive breast carcinoma is loss of epithelial phenotype in the tumor cells, which is characterized by detaching from nearby cells, degradation of basement membrane, invasion from surrounding tissues, and finally metastasis to other organs. The tumor's interplay with its stromal microenvironment in BC is a pivotal determinant of its progression from in situ carcinoma to metastatic and invasive advanced malignancy. As transformed cells degrade the stroma and basement membrane to invade the vessels, they also modify stromal signals towards stimulating angiogenesis, inflammation and tissue repair. During this process malignant cells secrete chemokines and growth factors that modify the stroma and alters the cell-extracellular matrix (ECM) interactions in favor of more efficient migration [3,4]. Tumor-secreted proteases such as matrix metalloproteases (MMPs) play an important role at tumor-stroma boundary environment. They degrade basement membrane and trigger migratory pathways. MMPs-mediated proteolysis creates cryptic sites in the collagen IV and laminin-5 (constituents of ECM) and promotes tumor cells migration [3,4]. Proteolysis of the basement membrane also induces the release of growth factors such as fibroblast growth factor (FGF) and insulin from ECM [5,6]. During the membrane shedding process, MMPs cleave several signaling molecules and release their ectodomains such as tumor necrosis factor (TNF-α) [7] and transforming growth factor (TGF)-β [8] from the cell membrane, which enhances the survival and migration of cancer cells [9]. The family of ADAM (A Disintegrin and Metalloprotease) proteases comprises 40 isoforms and shares multiple similarities with MMPs particularly in zinc-binding, disintegrin, and cysteine-rich domains. Similar to MMPs, ADAMs promote proteolysis of the basement membrane and proteins shedding from cell membrane. ADAMs also facilitate cell adhesion by their cysteine-rich domains [10] and their single transmembrane domains known as syndecans [11]. The cytoplasmic Src homology-3 binding domain of ADAMs induces cellular signaling through activation of Grb and Src proteins [12]. A disintegrin domain mediates binding of ADAMs to integrin molecules.

ADAMs family members are involved in remodeling of cell-surface, ectodomain shedding, regulation of growth factor availability and cell to cell and cell-matrix interactions in normal and pathological conditions such as Alzheimer's disease, rheumatoid arthritis, and cancer [13]. In 1993 it was reported that ADAM-11 gene locus at chromosome 17q21.3 goes under somatic rearrangements in 2 types of primary BCs [14]. In another research in 1999, enhanced mRNA and protein expression of ADAM-12 was recognized in infiltrating ductal BCs in comparison with equivalent normal breast tissues [15]. Since that time ADAMs' role in BC pathology has been widely studied and therefore the purpose of our review article is to provide an overview on the role of ADAMs in breast malignancy.

## 2 Structure and function of ADAMs

ADAMs are membrane-anchored glycoproteins and type-I transmembrane proteins that have broad biological functions such as stimulating intracellular signaling, cell adhesion, proteolysis, fertilization, migration, and cell fusion [16,17]. They also take part in tissue remodeling, organogenesis, extracellular matrix homeostasis, and inflammation [18,19]. Nearly fifty percent of the ADAMs have a common catalytic feature with metalloproteinases which allow them to be proteolytically active. The rest of ADAMs presumably participate in cell adhesion process [20,21] and just one third of ADAMs have the zinc binding common motif in the metalloproteinase domains [21]. ADAMs consist of metalloproteinase, cysteine-rich, proproteinase, epidermal growth factor-like, disintegrin, and cytoplasmic domains. ADAMs are structurally related to the snake venom reprolysins (SVMPs). The SVMPs degrade basement membrane constituents, such as fibronectin, laminin, and type-IV collagen and cause tissue hemorrhage. Some disintegrin domains in SVMPs consist of an integrin ligand sequence including RGD (arginine-glycine-aspartatic acid). SVMPs disintegrin domain can interrupt cell-matrix and cell-cell interactions that are integrin-mediated. ADAMs intriguing structural similarity to SVMPs suggests their ability to participate in proteolysis and cell adhesion in a diverse range of biological processes [15]. Both ADAMs and SVMPs belong to the reprolysin/adamalysin subfamily of zinc dependent metalloproteases [22]. Among the members of the metzincin subgroup and Zn^2+^-dependent protease superfamily, ADAMs and MMPs are largely involved in ECM remodeling and degradation. ADAMs and MMPs are implicated in tumor-promoting processes such as tumor growth, extravasation, stroma and basement membrane invasion, angiogenesis, and migration [12,23]. In fact, ADAMs are the only cell surface proteins that possess both adhesion and protease domains. Moreover, several ADAMs have cytoplasmic tails with SRC homology 3 binding sites, suggesting their probable interaction with cytoskeleton and intracellular signaling [24]. ADAMs are also mentioned with other names including MDCs which stands for metalloprotease/disintegrin/cysteine and cellular disintegrin [15].

Although it is shown that some specific ADAMs affect a variety of ECM proteins [25,26], but their major substrates are frequently membrane-bound proteins [16,17]. In fact, the most significant family of proteases that is involved in the modification and shedding of cell membrane's proteins appears to be ADAMs [27]. Multiple ADAMs have been shown to release growth factors, adhesion molecules, cytokines, receptors, and other proteins from the surface of the cells, a process known as ectodomain shedding [20]. Proteins ectodomains Shedding from tumors cells outer membrane results in production of cancer markers in serum. Different biological phenomena including cell proliferation, differentiation, survival and migration are mainly mediated and regulated through ectodomain shedding process [28]. This sheddase activity modifies cell surface receptors density and ligand stimulated cellular signaling cascades. Cysteine rich or disintegrin domains facilitate attachment to beta1 (b1), beta3 (b3) and alpha-V (αV) integrins [29] or syndecans [11] and impress cells to matrix and facilitate cell-cell interactions. These interaction are particularly important for tumor or inflammatory cells migration [30].

**Fig. 1:**
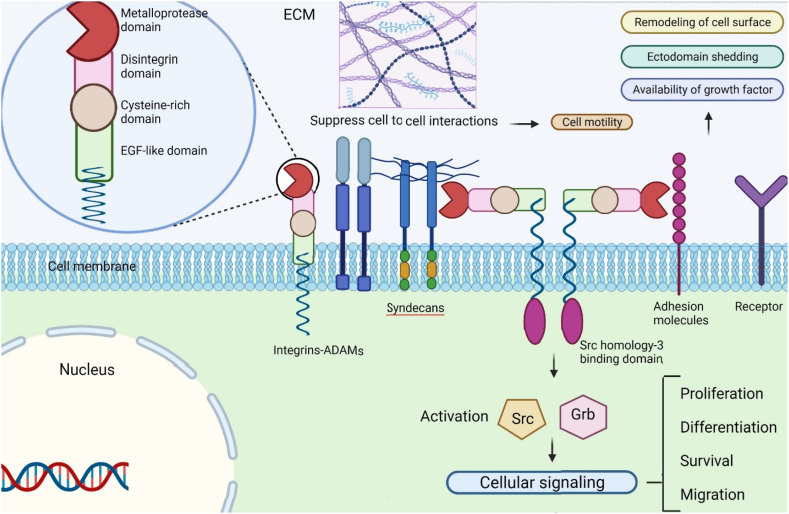
**Structure of ADAM molecules and their function in cancer induction.** ADAM molecules.

Modified expression and function of integrin molecules in carcinomas have been demonstrated in several studies [[31], [32], [33]]. Presence of integrins in ECM provide stabilized anchorage points and is supposed to need frequent changes as cancerous cells grow, variate morphology, and spread. Studies have shown that ADAMs are correlated with integrin molecules in the tissues [29,34] and they can perform Integrin shedding into ECM, along with tumor growth [35,36]. ADAM-integrin attachment suppresses a regular interaction between integrins and ECM, and as a result enhances cell motility and lead to increased spread of cancerous cells [30]. Accumulating evidences indicate that malignant tumors differentially express ADAMs which suggest that they may have a role in tumorigenesis (Fig. 1), (Table 1) [30].

**Table 1:** An overview of different ADAMs roles in breast carcinoma.

ADAMs	Role in Breast cancer	Mechanism	Reference
ADAM-8	Brain metastasis of primary Breast cancer promoting an invasive phenotype	Upregulating MMP-9 through ERK1/2 and CREB signaling, Protease Web/leukocyte mimicry of cancer cells through PSGL-1 cleavage interaction with β1 integrin and its localization and activation	[34] [2,37,38]
ADAM-9	Breast cancer development promotion of cell survival and invasion processes	Degrading TNF-α, fibronectin, gelatin, casein, c-kit ligand-1, p75 TNF receptor, and heparin binding epidermal growth factor/binding to the integrin and migration by laminin/processing EGFR-ligands cleaving precursor -proHB-EGF- to generatation HB-EGF and activation of AKT and STAT3 pathways	[30,39,40] [41,42]
ADAM-10	Breast cancer development and inducing drug resistance suppressing antitumor immune responses against cancer cell	releases HER2's extracellular domain and Elevation of p95/increasing GPNMB/ErbB signaling pathways proteolytic cleavage of PD-L1 and shedding from the cell surface	[[43], [44], [45], [46], [47]] [48]
ADAM-11	Breast cancer suppressor	–	[14,49]
ADAM-12	Malignancy induction in breast cancer Immunosuppression promotion	binding to heparin sepharose on cell surface/decreasing apoptosis of tumor cells and increasing apoptosis of stromal cells/NF-κB, TGF-β1/EGF and EGFR pathway/CD44^hi^/CD24^-/lo^ changing gene expression of TINs/PMN-MDSCs (tumor-infiltrating neutrophils/polymorphonuclear-myeloid derived suppressor cells)	[15,50,51,52,53] [54]
ADAM-15	tumor aggressiveness including cell growth and survival	E-cadherin sheddase/interacting with Tks5/Fish and Grb2 and extracellular signal regulated kinases/isoform specific manner association with Brk, the Src and Nck tyrosine kinases upregulation of the tight junction protein Claudin-1 through the activation of the PI3K/mTOR pathway	[20,55] [56]
ADAM-17	Breast carcinoma progression and malignancy suppressing antitumor immune responses against cancer cell	TGF-α converting enzyme/sheddase for HB-EGF, TGF-α, epiregulin, and amphiregulin/cleavage of TNF-α, TNF receptor, CD40, EGF receptor L, c-kit, p75NTR, c-fms, interleukin-1 receptor, interleukin-6 receptor growth hormone receptor, L-selectin, MUC1, vascular cell adhesion molecule-1, collagen VII, CX3CL-1, Notch, prion protein, and β-amyloid precursor/EGFR/HER2/neu pathway/Nectin-4 sheddase/proliferating cell nuclear antigens and urokinase plasminogen activator association/decreasing ADCC proteolytic cleavage of PD-L1 and shedding from the cell surface	[27,28,57,58,[59], [60], [61], [62], [63], [64], [65],[66], [67], [68], [69],^70^] [48]
ADAM-19	advancement of colony formation	Under the influence of H2BE76K transformation in histone/release of different development components such as neuregulin, heparin-binding epidermal growth factor, TNF-α, and TNF-related activation-induced cytokines	[71]
ADAM-22	endocrine resistant carcinomas progression/short disease-free survival/carcinoma reduction effect by a specific molecule induction (LGI1)	Regulated by SRC-1/binding to the neuropeptide LGI1	[72,73]
ADAM-23	Breast cancer regression migration of cancer cells	Interaction with avb3 integrin –	[24,74] [75]
ADAM-28	Proliferation induction in cancer cells	increased bioavailability of IGF-I by selective IGFBP-3 cleavage	[21]
ADAM-29	Breast malignancy induction	–	[76]
ADAM-33	tumor regression	DNA methylation leading to ADAM-33 gene silencing and tumor progression	[77]
ADAMTS9	angiogenesis inhibition and inhibition of the proliferation, colony formation, and invasion of breast cancer cells	by sequestering miR-513a-5p and thereby influencing cellular behaviors	[[78], [79], [80]]

## 3 ADAMs in breast cancer

### 3.1 ADAM8

ADAM8 is abundantly expressed in breast tumors when compared with normal breast tissue. it is related with a forceful phenotype and poorer patient prognosis [2]. ADAM8 expression is strongly associated with primary breast tumor formation and metastasis, suggesting that it plays a key role in the development of BC. Evidence suggests that ADAM8 positive tumor cells accumulate in the invasive zone of the tumor and its expression is maintained during metastasis [2,37]. In 2018, Conrad et al. reported increased expression of ADAM8 in primary BC which was associated with metastasis to brain. They indicated that ADAM8 contributes to transmigration through endothelium network and blood brain barrier. Moreover, they proved that the proteolytic feature of ADAM8 is owing to upregulating expression and activity of MMP-9. They also provided mechanistic evidences for the mentioned correlation. These evidences support this implication that in a cross talk manner metalloproteases regulate each other, which is termed as the “Protease Web”. They represented that ADAM8 reinforces two steps in brain metastasis process from breast tumor; the first step is “leukocyte mimicry” of cancer cells, which is through stimulating the cleavage of PSGL-1 by ADAM8. This cleavage modulates P-selectin/ligand PSGL-1 interactions. The second step is enhancing extracellular proteolysis by activated MMP-9 transcription through ERK1/2 and CREB signaling. It seems that correlated protease activity of MMP-9 and ADAM8 is one main mechanism underlying the brain metastasis. Clinical data shows that ADAM8 can be a proper predictor for metastasis [81]. *In vitro* analysis by Boyden chamber assay and 3D-Matrigel growth test have shown that, knocking down ADAM8 in triple-negative BC cell lines such as MDA-MB-231 and Hs578t leads to a decrease in their capacity to emigrate and attack through matrigel and forming branched colonies, respectively. In another *in vivo* assay, the ADAM8-deficient MDA-MB-231 cells were inoculated in a mouse orthotopic BC model. It was observed that tumor size was significantly decreased, circulating tumor cells were diminished, and the level of brain metastases recurrence was lowered significantly. These results indicate that ADAM8 has a significant role in tumor development and spread *in vivo*. Moreover, it was observed that in the ADAM8 deficient MDA-MB-231 tumors vessel formation is significantly reduced compared to MDA-MB-231 wild-type tumors. This shows that tumor angiogenesis is also restricted in the lack of ADAM8. *In vivo* observations were confirmed when endothelial cells *in vitro* were exposed to conditioned supernatants of ADAM8 knockdown, and they failed to stimulate the formation of vessel-like structures. Cell binding experiments using a fully formed layer of endothelial cells, which imitates the adhesion process to blood vessel walls during both intra- and extravasation, demonstrated the vital role of ADAM8 and its interaction with β1 integrin in the adhesion of BC cells to endothelial cells. ADAM8 has the ability to alter the adhesive characteristics of MDA-MB-231 cells towards endothelial cells by affecting the localization and activation of β1 integrin on the cell surface. As a result, this leads to a transformation in cell morphology, promoting an invasive phenotype [2,37,38]. ADAM8-dependent signaling through β1 integrin can trigger the release of miR-720, which plays a crucial role in sustaining the migratory and invasive characteristics of BC cells [82]. After attaching to the blood vessel, tumor cells perforate into the endothelium to gain access to the bloodstream or to reach a secondary organ. The increased presence of ADAM8 in BC cells notably enhances their migration through an endothelial layer, a process reliant on the cleavage of PSGL-1 by ADAM8 *in vitro*. Conversely, when ADAM8 was silenced or blocked, transmigration was effectively reduced. Transmigration necessitates the proteolytic degradation of tight junction proteins, which is further intensified through the regulation of MMP-9 expression via ERK1/2 and CREB signaling pathways at the transcriptional level. The significant and consistent association between ADAM8 and MMP-9 across 17 different BC cell lines serves as an evidence for the broad regulatory influence of ADAM8 on MMP-9's transcriptional activation [37]. Therefore, it is highly probable that the coordinated regulation of MMP-9 together with ADAM8 proteinase activity constitutes a critical mechanism in BC progression and metastasis. Consistent with the significant role of ADAM8 in BC development, the use of a therapeutic monoclonal antibody targeting the ADAM8 ectodomain has shown promising outcomes in an orthotopic BC mouse models. By administering intra-peritoneal injections of 0.5 mg/kg anti-ADAM8 antibody twice a week after tumor cell implantation in the mammary fat pad, substantial reductions were observed in the primary tumor size and burden, as well as in the number and size of brain metastases. Interestingly, the administration of anti-ADAM8 treatment to pre-existing tumors followed by a maintenance treatment for a duration of 5 weeks after tumor resection on day 15 exhibited a remarkable reduction in the metastatic capacity. This closely mirrors the clinical scenario encountered by BC patients where, initial diagnosis, neoadjuvant therapy and surgical resection take place. The findings suggest that such therapeutic intervention that target ADAM8 holds promise in mitigating metastatic progression post-surgery and offer a potential therapeutic strategy for BC patients [2].

### 3.2 ADAM9

Like other members of the ADAM superfamily, ADAM9 (the integrin binding metalloprotease) modulates extracellular and intracellular signaling via cell-matrix and cell-cell interactions modification ectodomain shedding of receptors on cell surface [39]. ADAM-9 proteolyze several substrates such as TNF-α, fibronectin, gelatin, casein, c-kit ligand-1, p75 TNF receptor, and heparin binding epidermal growth factor which is an effective mitogen for different cell types. In addition to proteolysis, ADAM9 is shown to have a role in cell adhesion by binding to integrins (e.g., on myeloma cells) and enhances cell migration by laminin, which is a constituent of basement membrane. ADAM9's ability to degrade specific ECM substrates and its role in cell adhesion suggest its probable intervention in cancer development [40]. There is also differential processing of ADAM9 in malignant and benign breast tissues. In addition, the expanded expression of the mature form (84 kDa) in the carcinomas positively correlated with certain BC prognostic factors such as HER-2*neu* protein levels and nodal status. Altogether, data suggest that this form of ADAM9 when enhanced, results in adverse outcomes in BC patients [40]. In another study, it is suggested that ADAM9 straightly involved in the cell-matrix interactions or by processing EGFR-ligands affect downstream signaling pathways [30]. Moreover, Sieuwerts et al. showed that in estrogen receptor positive primary BCs, ADAM9 and ADAM11 mRNA levels measurement seemed to be beneficial to diagnose BC recurrence in patients who have received tamoxifen therapy. High amounts of ADAM9 and ADAM11 were remarkably correlated with higher potency of tamoxifen therapy [83]. Transcripts of ADAM9 by alternative splicing process express ADAM9-L, a transmembrane protein and ADAM9-S, a secreted variant. In a study in 2010, specific antibodies of ADAM9-S and ADAM9-L were applied to detect both variants expression in BC tissues and cell lines. It was indicated that ADAM9-S by its metalloprotease activity elevates cell migration in BC, while ADAM9-L inhibits cell migration in a metalloprotease independent manner. ADAM9-L suppresses migration by a functional domain of disintegrin and integrin binding. Analysis revealed the expression of both isoforms in BC tissues and cell lines. Thus, relative amounts of membrane-anchored and secreted forms of ADAM9 are leading determinatives in aggressive migratory phenotypes manifestation and is correlated with BC development. This study implied that there are isoform-specific functions and for the first time disclosed a migration suppressor ADAM protein. These findings can contribute to small molecule inhibitors development for therapeutic interventions [39]. It was also indicated that silencing ADAM9 expression in MDA-MB-231 cell lines and subsequently production of ADAM9 knockdown clones of these BC cell lines suppressed the invasion of tumor cells. These suggest that this molecule plays a remarkable role in invasion and metastasis processes [84]. The role of ADAM9 is also investigated in lymphatic cancer cell dissemination and hematogenous in order to assist the design of novel therapeutic tools. RNA silencing approach was applied to study the effect of ADAM9 on the interaction between breast tumor cells and endothelial cells. It was observed that ADAM9 silencing reduced *in vitro* transendothelial cell migration. According to these results, ADAM9 has a role in the extravasation level of the metastatic cascade via both lymph and blood vessels [85]. In a study conducted in 2018, it was discovered that the NSD2 protein is highly expressed in tumors of triple-negative breast cancer (TNBC), and this elevated expression is associated with poor survival outcomes. Additionally, the study revealed that NSD2 plays a critical role in the regulation of TNBC cell survival, invasion, and tumor growth. It achieves this by directly controlling the expression and signaling of ADAM9 and EGFR. NSD2, functioning as a histone methyltransferase, specifically catalyzes di- and trimethylation of lysine 36 residues on the histone 3 tail (H3K36). NSD2 is recruited to the gene promoters of ADAM9 and EGFR, where it modifies histones through methylation, which ultimately influences their expression levels [86]. ADAM9 enzyme functions by cleaving the transmembrane precursor -proHB-EGF- to generate HB-EGF, which is a soluble ligand for the epidermal growth factor receptor (EGFR). The binding of HB-EGF to EGFR activates downstream signaling pathways, including AKT and STAT3, which are vital for cell survival and invasion. This mechanism ultimately facilitates the promotion of cell survival and invasion processes [41,42]. The study revealed that circ-ADAM9, a circular RNA, was significantly upregulated in both BC tissues and cells compared to control samples. Circ-ADAM9 was found to exert a crucial role in BC progression. Inhibition of circ-ADAM9 expression resulted in impaired proliferation, migration, and invasion of BC cells, while increasing radiosensitivity and apoptosis. Notably, when radiotherapy was combined with circ-ADAM9 inhibition, it exhibited significant inhibitory effects on tumor growth. The functional effects of circ-ADAM9 were found to be associated with miR-383-5p, a target of circ-ADAM9. Overexpression of miR-383-5p led to malignant behaviors and enhanced radiosensitivity in BC cells, and this effect was dependent on PFN2. Recent studies have provided evidence showcasing the involvement of profilin 2 (PFN2) in the regulation of actin polymerization and endocytosis processes [87,88]. Additional investigations have uncovered that circNINL/miR-921 axis serves as a mediator for the expression of ADAM9, since, ADAM9 is a direct target of miR-921. Moreover, it was demonstrated that ADAM9 has the ability to activate β-catenin signaling by interacting with E-cadherin [89].

### 3.3 ADAM10

In those BC cells with overexpressed HER2, the HER2 protein goes under proteolytic cleavage by ADAM proteases (mainly ADAM10). This process releases HER2's extracellular domain (ECD) into the serum and leaves out p95 (a cytoplasmic fragment) on tumor cells membrane which constitutively activates kinases. Elevation of ECD and p95 have been shown to be associated with poor prognosis and clinical outcomes as well as resistance to trastuzumab [43]. Therefore, HER2 sheddase inhibition can provide a novel approach for the treatment of patients with HER-2 positive BC [44,45]. As reported in the study performed by Codony-Servat et al., shedding of HER2 ECD can be suppressed by a wide range of metalloprotease inhibitors including TIMP-1 (tissue inhibitor of metalloprotease-1), TAPI and batimastat, suggesting that this protease can be an ADAM family member [46]. Moreover, combination therapy with low dosages of Herceptin and selective ADAM10 inhibitors reduced HER2 overexpressing cell lines' proliferation whilst those inhibitors that did not suppress ADAM10 had no such effect. It seems that ADAM10 is a major HER2 sheddase, and may provide novel approaches for the treatment of various cancers with active signaling of HER2 [44]. It is shown that suppressing cleavage of HER2 by INCB7839 (an ADAM10/ADAM17 inhibitor) decreases soluble HER2 ECD formation and also membrane p95 levels and subsequently renders tumor cells' response to treatment with trastuzumab [43]. INCB003619 is recognized to own selective and potent inhibitory effects against ADAM10 and ADAM17. This inhibitor efficiently suppresses HER-2 shedding in BC cell lines and their derived xenograft models that were HER-2 overexpressing/shedding. Moreover, INCB003619 can drastically boost trastuzumab's activity in the proliferation inhibition and signaling of HER- 2 overexpressing/shedding breast carcinoma cells. Furthermore, combination therapy with INCB003619 and trastuzumab potentiated the pro-apoptotic and antiproliferative activities of paclitaxel (a chemotherapeutic agent) [45]. It is also suggested that interrupting ErbB pathways by selective inhibition of metalloproteases from ADAM family can represent an approach for the treatment of human carcinomas where the ErbB signaling pathways activation have a role. This results in effective blockade of several ErbB pathways, leading to improved efficiency in the clinic [44]. In addition, GPNMB (Osteoactivin, DC-HIL) was recognized as an intervener in metastasis of BC to bone. It is also a prognostic representative of recurrence and an appealing therapeutic target for triple-negative BC patients. In addition, identifying ADAM10 as a sheddase of GPNMB has represented a molecular mechanism that inhibit ADAM10. ADAM10 sheddase activity on GPNMB is expected to increase expression of GPNMB on cell surface and hence potently enhances the efficiency of GPNMB targeting therapies [47]. The expression of programmed death ligand 1 (PD-L1) on the surface of tumor cells and its interaction with programmed cell death protein 1 (PD-1) on tumor-infiltrating lymphocytes play a role in suppressing antitumor immune responses. In breast tumors, PD-L1 expression is notably elevated in estrogen receptor-negative, progesterone receptor-negative and human epidermal growth factor receptor 2-negative (triple-negative) cancers. A recent study has demonstrated that in several triple-negative BC cell lines both exogenously and endogenously expressed PD-L1 undergo proteolytic cleavage by cell surface metalloproteases. This cleavage event generates a ∼37-kDa N-terminal PD-L1 fragment that is released into the surrounding media and a C-terminal PD-L1 fragment that remains associated with the cell but is efficiently eliminated through lysosomal degradation. This proteolytic processing of PD-L1 highlights a potential regulatory mechanism affecting its functional localization and have implications for therapeutic interventions targeting the PD-L1/PD-1 pathway in BC treatment. The ADAM10 protein is closely related to other members of the ADAM family, which are cell surface metalloproteases known to be involved in the cleavage of PD-L1. Interestingly, when cells are treated with ionomycin (a calcium ionophore that activates ADAM10), there is a substantial increase in the release of soluble PD-L1 into the surrounding media. This suggests that ADAM10 activation induced by ionomycin can enhance the proteolytic cleavage of PD-L1 leading to its shedding from the cell surface [48].

### 3.4 ADAM11

In 1993, it was reported that the ADAM11 gene which is located at 17q21.3 chromosome, somatically rearranges in two primary BCs. Originally, ADAM11 was recognized as a tumor suppressor gene candidate implicated in human breast carcinoma [14]. Based on its location on chromosome 17q21 within a heterozygosity region loss [90,91], ADAM11 has been suggested to be a suppressor for BC [14,49].

### 3.5 ADAM12

Human ADAM12 gene locates on 10q26 chromosome and encodes two different isoforms: ADAM12L (long transmembrane form) and ADAM12S (spliced secreted form). ADAM12S contains all of the extracellular domains but misses the cytoplasmic and transmembrane domains and alternately a strand of 33 distinct amino acids follows the EGF-like domain [92,93]. Moreover, Iba et al. found that ADAM12 is overexpressed in human tumor specimens and is located on the cell surface, playing role in cell-matrix or cell-cell adhesion. They explored recombinant polypeptides in the structure of cysteine rich domain of ADAM12 that are responsible for cell adhesion on the cell surface by binding to Heparin Sepharose [15]. ADAM12-L and ADAM12-S isoforms are also detected in the urine samples of patients with BC. It was reported that ADAM12 was found in the urine sample of BC patients in higher levels than controls. Moreover, detection with antibodies demonstrated that ADAM12-S active was present in the urine samples. The amounts of urinary ADAM12 were correlated with the stage of disease, as its level was increased in malignant BC. Accordingly, inspecting urinary ADAM12 can be useful in the development of non-invasive prognostic and diagnostic tests for breast carcinoma and other cancers [13]. Kveiborg et al. also reported that ADAM12 accelerates tumor development by decreasing apoptosis of tumor cells and increasing apoptosis of stromal cells. This dual governing cell survival role of ADAM12 is approved by another finding, which demonstrates that ADAM12 enhances nonneoplastic cells’ apoptotic sensitivity while increasing tumor cells resistance to apoptosis. Therefore, it seems that the tumor progressive role of ADAM12 is mediated through influencing apoptosis and not tumor cell proliferation [50]. In addition, investigation of ADAM12(S&L) and ADAM17 levels in laser-capture micro dissected (LCM) samples of BC showed that all of the three genes mentioned are upregulated in benign and malignant LCM tumor and malignant non-LCM samples. However, only ADAM12 is overexpressed in benign non-LCM tumor samples. The differences between LCM and non-LCM tumor samples were due to the influences of stroma on theses markers expression [57,94].

In another study, a cellular mechanism is identified for ADAM12 induction, which implicates NF-κB and its stimulated activation, by TGF-β1. TGF-β1 is shown to enhance ADAM12 mRNA expression in MDA-MB-231 BCE cells. A promoter element is responsible for TGF-β1–mediated induction of ADAM12. It is demonstrated that interaction between NF-κB and ADAM12 promoter and the elevated levels of NF-κB in BC cells leads to ADAM12 expression upregulation. Site-directed NF-κB element mutagenesis in the promoter of ADAM12 and NF-κB activity inhibition by Bay-11-7085 and MG-132 notably decreased TGF-β1-mediated enhancement of ADAM12 promoter-driven gene expression. Transfected cells with IκBαΔN, a dominant-negative mutant type of IκBα, which suppresses activation of NF-κB, remarkably decreased ADAM12 promoter-reporter transcription in TGF-β1-stimulated MDA-MB-231 BCE cells. Moreover, NF-κB overexpression stimulated the expression of ADAM12 in a dose dependent manner [51]. Furthermore, TNBCs depend on EGFR/ErbB1/HER1 signaling pathway to transform growth signals and induce cell proliferation. Dissolved EGF-like ligands are derivatives of their transmembrane progenitors by ADAM proteases. Results of the study performed by Li et al. provided novel insights into TNBC's biology. It is shown that ADAM12L is the only ADAM that its expression level is remarkably correlated with reduced remote metastasis-free survival duration. ADAM12L, HB-EGF, and EGFR were positively correlated with each other in TNBCs but not in ER-negative non TNBCs. It was demonstrated that ADAM12L's ectopic expression enhanced phosphorylation of EGFR in a mouse xenograft model of primary BC. Eventually, strong correlation was detected between the immunostaining levels of anti-ADAM12L and anti-phospho-EGFR in human breast carcinomas using tissue microarrays. These results suggested ADAM12L (as the initial protease) is responsible for EGFR activation in early stages of lymph node negative TNBCs [52]. In another study, it was implied that a breast carcinoma-associated mutation in ADAM12 modifies a prospective dileucine trafficking signal that can alter protein processing and cellular localization. In ADAM12 three somatic mutations that are cancer-associated were recognized. One mutation in the disintegrin domains and another in the metalloprotease domains were found to cause zymogen maturation failure, protein misfolding, and secretory pathway retention. p. L792F that is in the cytoplasmic tail of ADAM12 is the third mutation and is potentially remarkable, since it is located inside a di-leucine motif that is identified as a potential signal in cellular trafficking. Another study was also designed to investigate this mutation given its possible correlation with cancer and to define the di-leucine motif's role in ADAM12 trafficking. In mammalian cells expression of ADAM12p.L792F were shown to have quantitatively equivalent levels of expression and zymogen maturation compared with wild-type (WT) ADAM12, and also comparable cellular localizations. Levels of cell surface ADAM12 p. L792F and ADAM12 WT were similar and the mutant's rate and extent of internalization occurred identical for WT ADAM12. Additionally, in functional analysis assays no differences were shown in cell proliferations or ectodomain sheddase of EGF (an ADAM12 substrate for mutant ADAM12 and WT). According to these data, ADAM12 p. L792F mutation is improbable to drive cancer causing mutations in BC [95]. As discussed above, ADAM12 is overexpressed in human breast carcinomas and is a chemoresistance prognosticator in estrogen receptor-negative cancers. ADAM12 induction is through epithelial-to-mesenchymal transition, a characteristic of claudin-low breast carcinomas that is congested with markers of cancer stem cells (CSC). Another study was designed to investigate the role of ADAM12 in developing the CSC phenotype in breast carcinoma cells. High ADAM12 expressing cell populations had boosted CSC markers expression levels and a promoted capability of forming mammospheres. Knockdown of ADAM12 decreases cell migration, invasion, and anoikis resistance, and also compromises mammosphere formation. It was also reported that enrichment of CD44^hi^/CD24^-/lo^ and ALDEFLUOR ^+^ CSCs *in vitro* and also tumorigenesis in mice *in vivo* was diminished by knockdown of ADAM12. A remarkable overlap between EGFR-regulated genes and ADAM12 was identified through RNA sequencing. As a result, the basal activation of EGFR was lowered by ADAM12 knockdown. In addition, incubating cells with exogenouse EGF hindered lowering the CD44^hi^/CD24^-/lo^ cell population induced by ADAM12 knockdown. Altogether, these results implied that ADAM12 actively reinforces the CSC phenotypes in claudin-low expressing breast carcinoma cells by modulating the EGFR pathway [53]. Loss of ADAM12 in cancer cells has been found to enhance the chemotaxis of B cells *in vitro*. However, this effect can be reversed by inhibiting CXCR4, which is the receptor for CXCL12, or by using an anti-CXCL12 blocking antibody. Interestingly, the absence of ADAM12 in T11 cancer cells sensitizes tumors to combination therapy targeting PD1/CTLA4 immune checkpoints. However, acquired resistance to the therapy later replaces this initial responsiveness. Depletion of B cells in mice eliminates the improved response to immune checkpoint blockade in T11 tumors lacking ADAM12. Analysis of gene expression data in claudin-low TNBCs from the METABRIC patient cohort reveals significant inverse correlations between ADAM12 and gene expression profiles of various anti-tumor immune cell populations. Additionally, significant positive correlations have been observed between ADAM12 expression and the gene expression signature of TINs/PMN-MDSCs (tumor-infiltrating neutrophils/polymorphonuclear-myeloid derived suppressor cells) in TNBC. This suggests that ADAM12 may potentially play a role in promoting immunosuppression within the tumor microenvironment (TME) of TNBC [54].

### 3.6 ADAM15

ADAM15 is known to own secretase, disintegrin, and zymogen activities. ADAM15 is catalytically active and is normally expressed in primitive embryonic development, however, it is improperly expressed in diverse cancers, such as lung, prostate, and breast [55]. ADAM15 elevates extracellular sheddase of E-cadherin that is a soluble ligand of HER2/neu receptor, resulting in activation, proliferation, and increased motility. Targeted downregulating both HER2/neu and ADAM-15 function synergistically destroys BC cells. In this regard, a unique strand of guanine-rich DNA inside the promoter of ADAM15 was examined. This region contains seven conjunct sequences of three or more serial guanines that under superhelical stress, relaxes from duplex DNA and forms an intrastring structure of secondary G-quadruplex (G4). The G4 formation was examined within various sections of the ADAM15 promoter. Firm intrastring G4 formation was demonstrated to act as a biological quencher element. Characterizing the prominent G4 species that are formed inside the ADAM15 promoter can contribute to specified drug targeting and stabilizing, and allowing further novel targeted therapeutics development [55]. In addition, in a study designed by Ortiz et al. the ADAM15 gene copy number was demonstrated to be upregulated in BC cell lines. This effect was not displayed in mRNA levels; instead, the usage of alternative exons of ADAM15 appeared variable, resulting in aberrant ADAM15 mRNA isoform combinations in tumor cells. Modified alternative exon usage regulation of ADAM15 may be useful for cancer diagnosis since diverse combinations of its isoforms (derived from three alternative exons) can be easily examined with a PCR protocol [96]. Moreover, 4 isoforms of ADAM15 were examined in mammary carcinoma tissues. The ADAM15 variants were expressed in a differential manner in breast carcinoma tissues in comparison with normal tissues. Their expression was not associated with status of steroid hormone receptor, tumor size or grade, age, nodal status, menopausal status or Nottingham Prognostic Index. Although increased amounts of ADAM15A and ADAM-5B (two distinct isoforms) were correlated with poorer survival (relapse-free) in node negative patients. Enhanced ADAM15C isoform correlated with higher survival in node positive patients [20]. In MDA-MB-435 cell line, ADAM15A and ADAM15B expression had different impacts on cell morphology. While adhesion, invasion, and migration were elevated by ADAM15A, the expression of ADAM-15B decreased adhesion. The interaction abilities of the cytoplasmic domains of ADAM-15A/15 B/15C with Tks5/Fish and Grb2, the adaptor molecules, and extracellular signal regulated kinases are equivalent with each other. But they associate with Brk, the Src and Nck tyrosine kinases in an isoform specific manner. According to these data, differential ADAM15 variants expression determines tumor aggressiveness and clinical outcome by different interactions with intracellular pathways; especially ADAM15B due to owning broadest repertoire of interplaying partners is correlated with tumor malignancy [20]. In a study conducted in 2019, it was found that the expression of ADAM15 in BC cells resulted in the upregulation of the tight junction protein Claudin-1. This upregulation was mediated through the activation of the PI3K/mTOR pathway, which is involved in various cellular processes including cell growth and survival. The increased expression of Claudin-1 was found to be specific to certain isoforms of ADAM15 and dependent on its catalytic activity, indicating that the enzymatic function of ADAM15 plays a role in this regulatory mechanism. Interestingly, the effects on Claudin-1 expression varied depending on the specific cell background. Regarding cellular context influence, it is observed that there are differences in the impact of ADAM15 on Claudin-1 regulation between the MDA-MB-231 and MCF-7 cell lines. To further confirm the correlation between Claudin-1 and ADAM15, experiments were conducted on T47D cells which are known to have elevated levels of both proteins. The results of these experiments provided additional evidence supporting the dependence of Claudin-1 expression on ADAM15 Moreover, it was observed that ADAM15 co-localizes with Claudin-1 at cell-cell junctions. This co-localization is believed to be facilitated by the interaction of ZO1/2 SH3 domains with the intracellular domain (ICD) of ADAM15 [56].

### 3.7 ADAM17

One of the sheddases that has been extensively studied is ADAM17. It is also termed as TACE (TGF-α converting enzyme) [58],since, it was originally recognized by its capability to cut off membrane bound TNF- α from its progenitor [97,59]. ADAM17 is the main sheddase for HB-EGF, TGF-α, epiregulin, and amphiregulin [57,[60], [61], [62]] and it was demonstrated to be elevated in human breast carcinomas [97]. ADAM17 is also implicated in several biological procedures and cleaves plenty of substrates such as TNF-α, TNF receptor, CD40, EGF receptor L, c-kit, p75NTR, c-fms, interleukin-1 receptor, interleukin-6 receptor growth hormone receptor, L-selectin, MUC1, vascular cell adhesion molecule-1, collagen VII, CX3CL-1, Notch, prion protein, and β-amyloid precursor [59,[63], [64], [65]]. Mutations that inactivate ADAM17 reveal its significance during development [36].

Recognition and verification of a targeted treatment for TNBCs that is negative for progesterone receptors, estrogen receptors, and HER2 amplification is an essential problem in BC therapy. EGFR gene is one of the most validated drivers for TNBC. Normally, EGFR is activated following the release of ligands such as TGFα by two ADAMs; ADAM10 and ADAM17. In a study, antitumor effects of an ADAM17 antagonizing monoclonal antibody were investigated in TNBC *in vitro*. Results showed that targeting ADAM17 with D1 (A12) (a humanised monoclonal antibody) inhibits TGFα release, decreases proliferation, growth, migration and invasion and therefore, it can have anticancer activities in TNBC cells [98]. Moreover, considering that ADAM17 is a major modifier of EGFR signaling, the inhibitors of ADAM17 are hoped to be specifically useful in cancers that are associated with EGFR/HER2/neu pathway and in tumors showing resistance to tyrosine kinases or trastuzumab due to excessive formation of ligands, including TGF-α. Therefore, implementing such inhibitors along with antagonizing antibodies in combination therapy is also useful [[66], [67], [68], [69]].

Nectin-4, which is a Serological Marker for BC, is also one of the substrates of ADAM17. Fabre-Lafay et al. found that ADAM17's active form is upregulated in BC samples, implying that ADAM17 can shed Nectin-4 *in vivo* [28]. As discussed above, ADAM17 is a significant HER family receptors transactivator, specifically EGF receptor [[99], [100], [101], [102]]. EGFR plays a crucial role in mitogenesis, angiogenesis and cell migration and is currently receiving intensive attention for anticancer therapies [103]. In fact, following stimulation of G-protein receptor, release of particular EGFR ligands through ADAM17 has been proved to trigger transactivation of EGFR and increases migration and mitogenesis of various cell lines *in vitro* [[99], [100], [101], [102]]. Altogether, these findings suggest that ADAM17 stimulates cancer progression. It was also observed that upregulation of ADAM17 in MCF-7 cells enhances proliferation and invasion *in vitro*, while reduction of ADAM17 in MDA-MB-435 cells declined proliferation and invasion. Moreover, in primary cancers, both isoforms of ADAM17 associated remarkably with amounts of proliferating cell nuclear antigens and urokinase plasminogen activator. Findings support this notion that ADAM17 is implicated in breast carcinoma progression [27]. In another study, it was reported that, ADAM17 expression notably enhances in high-grade tumors in comparison with low-grade ones and did not relate to lymph node metastasis, estrogen receptor status, and tumor size. In patients with high expression of ADAM17 the overall survival was significantly shorter compared with low expressing ones. Remarkably, ADAM17's prognostic impact was independent from other conventional prognostic agents for BC. Further findings revealed a significant correlation between elevated levels of ADAM17 and poor outcome in BC patients and consequently provided further motivations for applying ADAM17 as a cancer treatment target [97]. Moreover, major histocompatibility complex class I (MHC I) molecules naturally process ADAM17 and this member of ADAM family can potentially be used as target antigen in immunotherapeutic strategies against prostate, ovarian, and BCs. Characterization of this molecule as an immunotherapy antigen suggests procession of vaccine strategies employing it [104]. In addition, since ADAM 17 is the main sheddase of EGFR-ligands and TGF-α from a compartment of a late secretive pathway [105], combination therapy is possible by combining EGFR pathway inhibition by small molecules along with immunotherapical treatment against ADAM17 [104]. Moreover, it was demonstrated that suppression of ADAM17 has the potency to become a NK cell based immunotherapy in cancer via enhancing the purity of extended NK cells and increasing antibody dependent cellular cytotoxicity (ADCC) against trastuzumab in the cell lines of BC [70]. In addition to ADAM10, ADAM17 has also been identified as an enzyme involved in the cleavage of PD-L1. When cells are treated with a specific activator of ADAM17 called phorbol 12-myristate 13-acetate (PMA), there is a significant increase in the release of soluble PD-L1 into the surrounding media. This suggests that both ADAM10 and ADAM17 play crucial roles in regulating the PD-L1/PD-1 pathway which in turn impacts anti-tumor immunity. Understanding the mechanisms by which these ADAM proteases influence PD-L1 cleavage can have significant implications, particularly in triple negative BC and the development of therapeutic strategies targeting this pathway [48].

### 3.8 ADAM19

The later recognizable proof of the H2BE76K transformation in histone H2B in different cancer types has revealed a novel category of oncohistones. The H2BE76K alteration debilitates the stability of histone octamers and drives gene expression modifications and colony formation progression. Within the context of BC, a study was conducted where the H2BE76K transformation were induced in MDA-MB-231 BCE cells. Upon analyzing the gene expression patterns, it was observed that genes upregulated within the H2BE76K mutant cells were enriched for the E76K mutant form of H2B and were participating in cell attachment and multiplication pathways. Surprisingly, one of the target genes affected by H2BE76K transformation was recognized as ADAM19, suggesting a potential inclusion of H2BE76K in controlling ADAM19 expression. ADAM19 is known as adamalysin 19, and a signal sequence pro-domain, a metalloproteinase domain, a disintegrin domain, a cysteine-rich domain, an epidermal development factor-like domain, a transmembrane domain, and a cytoplasmic domain form different domains of this protein. This cell surface glycoprotein act as an endopeptidase and is capable of cleaving extracellular matrix proteins. ADAM19 also plays a part in the release of different development components and cytokines. It is included within the discharge of neuregulin, heparin-binding epidermal growth factor, TNF-α, and TNF-related activation-induced cytokines [71].

### 3.9 ADAM22

ADAM22 (a non-protease component of ADAM family) was recognized as a direct target of SRC-1 which is ER independent. SRC-1 was affirmed as an ADAM22 regulator in molecular, cellular and *in vivo* experiments. As mentioned, its expression is driven by SRC-1 that is a coactivator protein, in response to treatment with tamoxifen in the resistant state [72,73]. It was observed that ADAM22 is involved in cellular differentiation and migration and its concentration was extended in endocrine resistant carcinomas rather than endocrine sensitive ones in human BC mouse xenograft models. Clinically, ADAM22 was observed to act as an independent prognostic factor of short disease-free survival. It has been proposed that SRC-1 shifts steroid-responsive cancers to a steroid resistant status in which ADAM22 as the SRC-1 target gene has a crucial role, expressing it as a target of prognosis and a therapeutic drug that can contribute to the improvement of endocrine-resistant BC treatment. A new ER-independent objective of SRC-1 is recognized. This disintegrin functions in endocrine dependent tumor metastasis brings up new alternatives as a treating target [72]. In addition, the neuropeptide LGI1 has been shown to bind ADAM11, ADAM22, and ADAM23 in the nervous system. This molecule has a tumor suppressing function. Besides, LGI1 plays a role in reducing migration and impairing proliferation. LGI1-based therapies can contribute to a building block for prospective treatments in ADAM22-positive breast carcinoma [73].

### 3.10 ADAM23

ADAM23 displays the common structure of the ADAM family components, despite of the metalloprotease domain that is inactive, which suggests that it is involved exclusively in the cell adhesion [24]. It was demonstrated that ADAM23 in an RGD-independent fashion interacts particularly with avb3 integrin [74]. Costa et al. measured the level of mRNA expression and methylation state of the fifty upstream regions of ADAM23 gene in several cell lines of breast carcinoma and primary cancers. Promoter hypermethylation of ADAM23 was observed in cancers that was strongly correlated with declines in expression levels of both of the mRNA and protein. It is worth reporting that primary BCs with a more developed grade exhibited a higher methylation degree, expressing that this adhesion molecule (ADAM23) is downregulated during BC progression [24]. The diminished methylation of the SNAI2 gene in tumors with histologic grade 3 proposes a novel role for this epithelial-mesenchymal transition (EMT) gene in cancer cell dedifferentiation. This finding indicates that SNAI2 contributes to the loss of cellular differentiation observed in high-grade tumors. On the other hand, variances in ADAM23 methylation profiles among BC patients with different routes of cancer cell dissemination suggest that ADAM23 plays a role in hematogenous spread, which involves the migration of cancer cells through the bloodstream. These observations highlight the potential involvement of SNAI2 and ADAM23 in important processes related to cancer progression and metastasis [75].

### 3.11 ADAM28

In 2006 Mitsui et al. reported that active form of ADAM28 is predominately expressed by tumor cells inside carcinoma tissues. A direct relevance was found between levels of mRNA expression in carcinoma cells and their proliferative activity. Treatment of BC cells (MDA-MB231) which express ADAM28 with insulin-like growth factor-I (IGF-I) enhances proliferation of cells. IGF binding protein (IGFBP)-3 cleavage and IGF-I signaling were significantly suppressed by anti-ADAM28 antibody or ADAM inhibitor treatment. Using small interfering RNA and down-regulating expression of ADAM28 in MDA-MB231 cells remarkably diminish IGFBP-3 cleavage, cell proliferation, and mice xenografts growth. Taken together, they found that ADAM28 is overexpressed in its activated form within human BC tissues by tumor cells and claim that ADAM28 is implicated in cell proliferation via increased bioavailability of IGF-I, which is released from the complex of IGF-I/IGFBP-3 by selective IGFBP-3 cleavage [21].

### 3.12 ADAM29

ADAM29 is implicated in numerous significant physiological processes. Recent researches have shown this molecule to be a susceptibility locus with features of a risk factor of human breast carcinoma under genome-wide importance. Its overexpression was found in breast carcinoma tissues in comparison with normal ones. ADAM29 expression and its mutations in variant domains remarkably affected BC cells proliferation, migration and invasion *in vitro*. Therefore, ADAM29 may be considered as a prognostic factor of BC, and also a novel candidate molecule to be utilized as a therapeutic target [76].

### 3.13 ADAM33

Downregulation of ADAM33 by promoter hypermethylation was reported in 2009. This seemed to be relevant with tumor development and metastasis in human BC. In the study done by Seniski et al. the implication of ADAM33 in BC was observed and the regulation of ADAM33 gene by epigenetic mechanisms such as DNA methylation was investigated in breast tumors. Analysis of ADAM33 expression in BC cell lines uncovered gene silencing in 65 % of cancer cell lines. They also reported that gene silencing is because of ADAM33 promoter hypermethylation. ADAM33 promoter hypermethylation was also observed in the samples derived from 40 % of primary BCs. In conclusion, these results proposed ADAM33 gene as a tumor suppressor that can be a beneficial molecular marker of breast invasive lobular cancer [77]. The ADAM33 gene (coding a transmembrane glycoprotein) is implicated in cancer progression and undergoes changes in cell adhesion. Studies indicate that hypermethylation of intron 1 within the ADAM33 gene is associated with increased ADAM33 expression and a reduced risk of BC. Notably, bisphenol A (BPA) and phthalates (known to possess epigenetic toxicity) have diverse effects on BC risk. Phthalate metabolites, including MEHHP, MECPP, MEOHP, and Σ4MEHP, appear to have protective effects in reducing BC risk. Conversely, we discovered a potential association between increased urinary concentration of BPA and BC. Furthermore, MEP and MBP, both phthalate metabolites were negatively correlated with ADAM33 expression [106].

### 3.14 ADAMTS

The ADAMTS gene encodes an enzyme belonging to the ADAMTS protein family which includes various protein modules such as a propeptide region, a metalloproteinase domain, a disintegrin-like domain, and a thrombospondin type 1 (TS) motif. Different family members differ in the number of C-terminal TS motifs and have unique C-terminal domains. The ADAMTS proteins have been associated with proteoglycan cleavage, regulation of organ shape during development and angiogenesis inhibition. The ADAMTS9 gene is located on chromosome 3p14.3-p14.2, a genomic region that is frequently lost in hereditary renal tumors. In 2020, Fang et al. demonstrated that the long non-coding RNA (lncRNA) ADAMTS9-AS1 has the ability to inhibit the proliferation, colony formation, and invasion of BC cells. It does so by acting as a sponge for miR-513a-5p, a microRNA molecule known to be involved in cancer regulation. This finding suggests that ADAMTS9-AS1 plays a role in suppressing BC progression by sequestering miR-513a-5p and thereby influencing cellular behaviors [[78], [79], [80]].

## 4 Conclusion

ADAM family members seems to have significant functions in cancer progression through their sheddase activity and modifying adhesion of cell/cell or cell/ECM. Due to capability of performing both tasks, some components of this family seem to play a crucial role in BC development and determine the progressiveness of tumor. Accordingly, modulating these transmembrane glycoproteins can be a promising approach in treating BC and they can also be exploited as prognostic and diagnostic markers [107]. As reviewed above, some of ADAMs induce proliferation, tumor growth, migration, metastasis, and invasion in BC. It was also shown that modulation of ADAMs chemosensitizes tumor cells towards chemotherapeutics such as Trastuzumab [43,68]. Therefore, it is recommended to study the effects of targeting ADAMs on chemoresistance towards other drugs since resistance to chemotherapeutics in many occasions is due to upregulation of the broad spectrum of ADAMs substrates. Hence, targeting ADAMs could dually benefit the treatment process by attenuating the tumor cells and sensitizing them to drugs applied. Owing to the tumor growth suppressing observations by targeting ADAMs, combination of ADAMs inhibition with radiotherapy can be effective. It is suggested to determine exact effects of each member in breast oncogenesis and also explore the implications of other members of ADAMs superfamily rather than the ones discussed and studied in BC progression. Since there are almost 40 members of them recognized [10]. We assume that ADAMs might also have roles in BC progression and it is beneficial to investigate and determine their mechanism of action to design more qualified combination therapies. It is also recommended to study and explore the isoforms of every single ADAMs and their distinctive functions and implications in cancer development, in view of the fact that some of ADAMs reviewed above have been recognized to have isoforms that do not share same functions in cancer. We also suggest inspecting the mechanisms underlying tumor-suppressing effects of some of the ADAMS such as ADAM-11 and ADAM-33, because despite their genetic basis of actions, their induced molecular pathways that appoint the suppressing effect has not been clear. The relation between HER2 signaling pathway and some ADAM molecules such as ADAM10 and ADAM17 have been studied [44,45,67] but it is recommended to broadly investigate the cross talk between them (with another ADAMs) and also study the correlations of ADAM molecules with other hormones such as estrogen induced signaling pathways. In order to reduce systematic side effects through targeting more specifically, it is suggested to combine ADAMs targeting with nano-based delivery methods and determine the best nano carrier for different ADAM modulators [83,108]. It is also worth investigating ADAMs modulators efficacy such as INCB7839 and [43,68] *in vivo* and clinical trials along with ongoing *in vitro* experiments. In addition, it can be useful to investigate their involvements and the efficiency of their modulators in different stages and types of BC. At last, it is worth mentioning that development of more efficient ADAM modulators and targeting them in combination with other therapeutic approaches can be useful.

## Conﬂict of interest statement

None of the authors has any conﬂict of interest to declare.

Consist of Metalloprotease domain, Dsintegrin domain, Cysteine-rich domain, and EGF-like domain.

ADAMs connect to integrins and prevent interaction of them with ECM that results in cell mobility. ADAMs could also cleavage some surface molecules and inhibit them that lead to ectodomain shedding, remodeling of cell-surface and availability of growth factors. Some of ADAM molecules also have cytoplasmic domains called Src homology-3 binding domain that activates Src and Grb resulting in cell signaling activation.

## Data availability statement

All data associated with this manuscript are available as a part of the manuscript and no additional source data are required.

## CRediT authorship contribution statement

**Sepideh Aliniaye Navasatli:** Writing – original draft. **Saeed Niazi Vahdati:** Validation. **Tahura Fayeghi Arjmand:** Writing – review & editing, Writing – original draft. **Hossein Behboudi:** Writing – review & editing, Writing – original draft, Supervision.

## Declaration of competing interest

The authors declare that they have no known competing financial interests or personal relationships that could have appeared to influence the work reported in this paper.
